# Senescence and senolytics in cardiovascular disease: Promise and potential pitfalls

**DOI:** 10.1016/j.mad.2021.111540

**Published:** 2021-09

**Authors:** W Andrew Owens, Anna Walaszczyk, Ioakim Spyridopoulos, Emily Dookun, Gavin D. Richardson

**Affiliations:** a Biosciences Institute, Newcastle University, Newcastle upon Tyne, NE1 3BZ, UK; b Atelerix Ltd, Newcastle upon Tyne, UK; c The James Cook University Hospital, Middlesbrough, UK

**Keywords:** Ageing, Atherosclerosis, Cardiovascular, Heart failure, Inflammation, Remodelling, Senescence, Senolytic

## Abstract

Ageing is the biggest risk factor for impaired cardiovascular health, with cardiovascular disease being the cause of death in 40 % of individuals over 65 years old. Ageing is associated with an increased prevalence of atherosclerosis, coronary artery stenosis and subsequent myocardial infarction, thoracic aortic aneurysm, valvular heart disease and heart failure. An accumulation of senescence and increased inflammation, caused by the senescence-associated secretory phenotype, have been implicated in the aetiology and progression of these age-associated diseases. Recently it has been demonstrated that compounds targeting components of anti-apoptotic pathways expressed by senescent cells can preferentially induce senescence cells to apoptosis and have been termed senolytics. In this review, we discuss the evidence demonstrating that senescence contributes to cardiovascular disease, with a particular focus on studies that indicate the promise of senotherapy. Based on these data we suggest novel indications for senolytics as a treatment of cardiovascular diseases which have yet to be studied in the context of senotherapy. Finally, while the potential benefits are encouraging, several complications may result from senolytic treatment. We, therefore, consider these challenges in the context of the cardiovascular system.

## Highlights

• Studies have demonstrated that senescence contributes to the pathophysiology of several age-related cardiovascular diseases.
• Senolytics eliminate senescent cells in cardiovascular tissues and prevent or reverse disease progression.
• While this data is encouraging, questions remain regarding the potential short and long-term detrimental effects of senolytic mediated apoptosis.

## 1 Cardiovascular ageing and disease

Ageing is the biggest risk factor for impaired cardiovascular health, over 75 % of Americans between 60–79 and nearly 90 % of over 80-year-olds present with cardiovascular disease (CVD) (Virani et al., 2021). Age-associated CVDs include atherosclerosis, coronary artery stenosis, myocardial infarction (MI), thoracic aortic aneurysm (TAA), valvular heart disease (VHD) and a progressive decline in cardiac function (Childs et al., 2017; Olivieri et al., 2013). It is therefore unsurprising that CVD is the cause of death in 40 % of individuals over 65 years old (North and Sinclair, 2012). As our population continues to age the incidence of CVD will continue to rise to create an even larger global health and economic burden.

Studies have long linked senescence with CVD. Cellular senescence is defined as the irreversible loss of division potential in somatic cells. While senescence can be a potent anti-tumorigenic mechanism, recent studies have indicated that it is a major contributor to age-dependent tissue dysfunction (Campisi and d’Adda di Fagagna, 2007). Senescent cells accumulate in the majority of organ tissues with age (Herbig et al., 2006; Wang et al., 2009), as well as in several age-related diseases (Campisi and d’Adda di Fagagna, 2007; Gonzalez et al., 2008). Senescent cells are thought to contribute to ageing not only as a result of cell cycle exit but also via a pro-oxidant phenotype and the secretion of a plethora of pro-inflammatory cytokines, chemokines, matrix metalloproteinases and growth factors termed the senescence-associated secretory phenotype (SASP) which can promote senescence in surrounding cells (the bystander effect) (Nelson et al., 2012). The identification of senescence cells is challenging, particularly in vivo, as to date no unique markers have been identified that explicitly allow the detection of all senescent cells (Sharpless and Sherr, 2015) and the senescent phenotype is heterogenous (de Magalhaes and Passos, 2018). While cell cycle arrest has been used for senescent cell detection, post-miotic cells including cardiomyocytes (CM) (Anderson et al., 2019), neurons (Hewitt et al., 2012), osteocytes (Farr et al., 2016) and adipocytes (Baker et al., 2011; Minamino et al., 2009) can also become senescent. As such, a multimarker combinatorial approach is required for the accurate assessment of cellular senescence. In vivo this is commonly performed using classical hallmarks of senescence including senescence-associated β-galactosidase (SA-β-Gal) activity, as a result of increased lysosomal content, the expression of cyclin-dependent kinase inhibitors including p21**^Cip^**, p16**^Ink4a^**, and p53, the presence of DNA damage or by the presence of critically short telomere length (de Magalhaes and Passos, 2018; González-Gualda et al., 2021).

As early as 2008 mice models of induced senescence have been used to demonstrate the link between accumulated cellular senescence and myocardial remodelling with cardiac dysfunction. Mice that lack telomerase reverse transcriptase (TERT**^−/−^**), the enzymatic component of telomerase, exhibit heritable telomere shortening (Blasco et al., 1997) and demonstrate telomere dysfunction after 3–6 generations depending on the genetic background (Lee et al., 1998). TERT**^-/-^** mice demonstrate accelerated ageing and a plethora of age-related disease phenotypes including a decreased life span, reduced body size and weight, hair greying and loss, infertility and testicular atrophy, spleen atrophy, immunosenescence, and impaired wound healing (Wong et al., 2008). The hearts of late generation TERT**^-/-^** mice also demonstrate increased expression of the senescence-associated tumour suppressor protein p53 in the CM population and functional changes including left ventricular (LV) diastolic dysfunction, decreased CM number and increased CM hypertrophy (Leri et al., 2003) all of which are hallmarks of myocardial ageing in humans (Villari et al., 1997). Similarly, genetic conditions resulting in an accumulation of cellular senescence and accelerated ageing phenotypes are associated with CVD. Sufferers of dyskeratosis congenita a hereditary disorder characterised by mutations in genes that encode either TERC or TERT (Armanios et al., 2005; Vulliamy et al., 2001) demonstrate an increased prevalence of CVDs including increased cardiac fibrosis and dilated cardiomyopathy (Ballew and Savage, 2013; Khincha et al., 2017; Lina et al., 2008; Vulliamy et al., 2004).

**Fig. 1:**
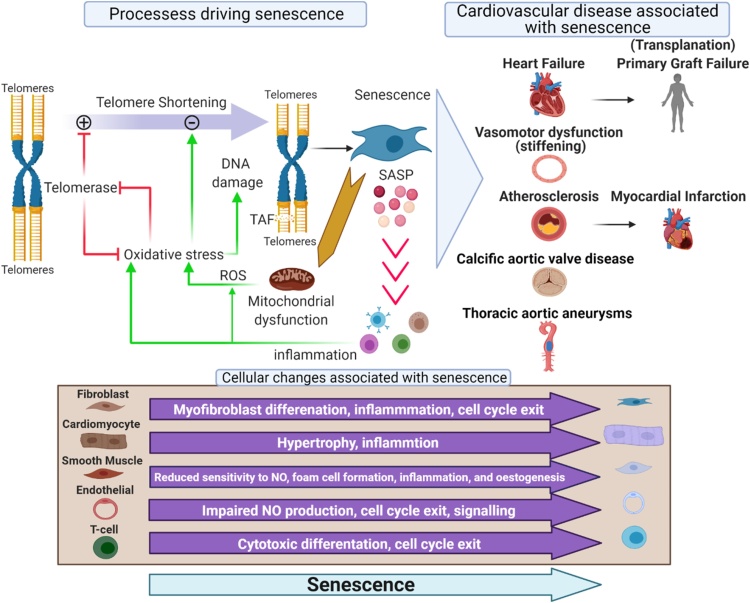
Senescence and cardiovascular disease. *Processes driving senescence*. Cellular senescence is induced by telomere dysfunction and DNA damage. This occurs as a result of telomere shortening during proliferation and the associated DNA synthesis, but also as a result of oxidative stress. Mitochondrial dysfunction increases ROS which accelerates telomere shortening via 8-oxodG formation, inhibits TERT transcriptional activity harming telomere homeostasis and leads to the formation of persistent telomere-associated double-stranded DNA breaks. Telomere dysfunction induces cellular senescence via activation of the DNA damage response. As TERT participates in the control of mitochondrial ROS generation, TERT inhibition also contributes to increased ROS and further intracellular oxidative stress. *Cellular changes associated with senescence.* Once senescent, cells promote a cascade of senescence via the senescence-associated secretory phenotype which induces a bystander effect and increases inflammation and oxidative stress. The senescent phenotype is cell dependant but drives phenotypic changes associated with the initiation and progression of multiple cardiovascular diseases, including but not limited to those presented.

During ageing cardiovascular tissues accumulate senescence by way of several cell type-dependent mechanisms which include mitochondrial dysfunction, increased reactive oxygen species (ROS), DNA damage and telomere dysfunction (Anderson et al., 2018) which induces phenotypic changes associated with the initiation and progression of multiple cardiovascular diseases (Fig. 1). Growing evidence indicates that, as with the models and genetic diseases described above, these phenotypically altered naturally occurring senescent cells trigger or exacerbate the onset and progression of numerous CVDs (Anderson et al., 2018; Dookun et al., 2020a). This notion is best supported by studies that have utilised transgenic models in which p16**^Ink4a^** expressing senescent cells can be specifically eradicated (Baker et al., 2016, 2011; Demaria et al., 2014a). These studies demonstrate that elimination of senescent cells and a reduced cardiovascular senescence cell burden is associated with the delay, prevention, or reversal of characteristics of a variety of CVDs (Anderson et al., 2019; Baker et al., 2016, 2011; Childs et al., 2016; Lewis-McDougall et al., 2019).

Now with the identification of compounds termed senolytics, due to their ability to preferentially induce apoptosis in senescent and not non-senescent cells, groups have begun to test the therapeutic potential of pharmacological senescence elimination to prevent or reverse age-related CVD. In this review, we highlight those studies that suggest potential clinical applications of senotherapies for the treatment of CVD. Furthermore, we will discuss the potential pitfalls that may hinder the translation of the preclinical studies - given the available literature regarding the undesirable on target and off-target side effects and toxicity profiles of senolytic compounds (Blagosklonny, 2018; Malavolta et al., 2018; Medeiros et al., 2018; Pignolo et al., 2021), including those in the present issue of this journal, our review will focus specifically on the pitfalls relevant to the cardiovascular system as opposed to more generic challenges.

## 2 Senolytics

Senescent cells have active DNA damage responses, increased mitochondrial dysfunction and mitochondrial membrane permeability, increased ROS production, heightened metabolic flux, and produce an inflammatory SASP creating hostile internal and external microenvironments, (João F Passos et al., 2010; João F. Passos et al., 2007; Y. Zhu et al., 2015) yet despite this, senescent cells remain viable and accumulate in tissues with age. This may in part be due to the fact that senescence is associated with increased survival and resistance to apoptosis (E. Wang, 1995). Based on these observations Zhu et al. hypothesised that targeting components of these pro-survival networks would selectively eliminate senescent cells (Y. Zhu et al., 2015). Following the demonstration that siRNA mediated inhibition of components of the pro-survival networks of senescent cells preferentially reduced the viability of senescent cells, but not proliferating or quiescent, differentiated cells, Zhu et al. targeted these pathways pharmaceutically (Y. Zhu et al., 2015). These and subsequent investigations have identified compounds that target components of anti-apoptotic pathways can promote senescent cell apoptosis in vitro and result in systemic senescent cell reduction in vivo (Kirkland & Tchkonia, 2017; Short, Fielder, Miwa, & von Zglinicki, 2019; Xiong et al., 2015; Y. Zhu et al., 2015). These compounds have been termed senolytics (Y. Zhu et al., 2015). The survival pathways targeted include Bcl-2 family members, p53/p21Cip, ephrins (EFNB1 or 3), the phosphatidylinositol-4,5-bisphosphate 3-kinase (PI3K), plasminogen-activated inhibitor-1 and 2 (PAI1 and 2) and hypoxia-inducible factor-1α.

**Fig. 2:**
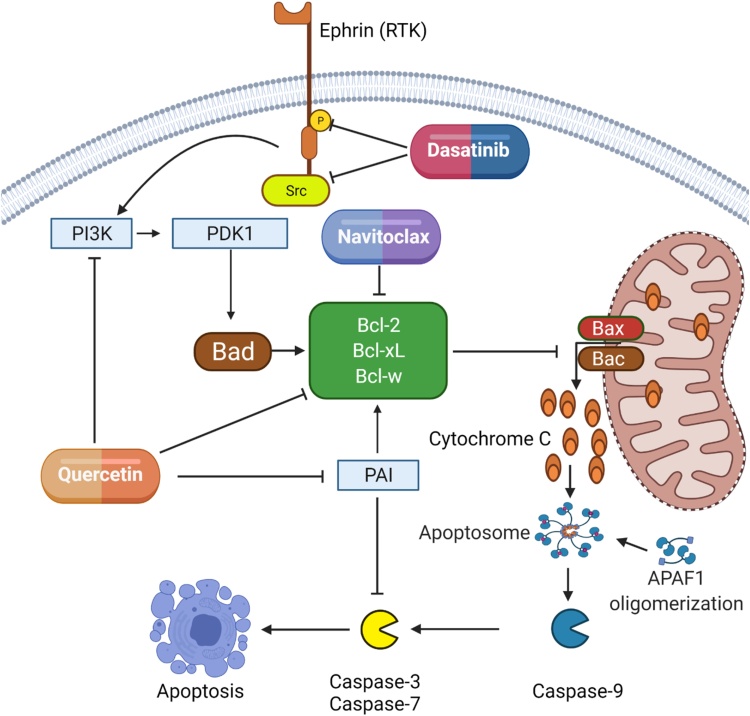
Navitoclax, dasatinib and quercetin promote apoptosis through the inhibition of pro-survival proteins. During apoptosis, Bax and Bak generate outer mitochondrial membrane pores allowing mitochondrial outer membrane permeabilisation (MOMP) and the release of cytochrome-C. Cytochrome-C and apoptotic protease activating factor-1 (APAF1) form the apoptosome which recruits and activates pro-caspase-9 which in turn initiates the caspase cascade which includes caspases 3 and 7. Dasatinib inhibits receptor tyrosine kinases (RTK) such as ephrin B and non-receptor tyrosine kinases including Src. These tyrosine kinases regulate the pro-survival PI3K-PDK pathway and downstream Bcl-2 family protein activity inhibiting Bax and Bak pore formation and thereby apoptosis. Similarly, quercetin is an inhibitor of both the PI3K-PDK pathway and Bcl-2 proteins, however, quercetin also inhibits PAI, a protein that could promote cell survival via the upregulation of Bcl-2 expression and as a result of complex formation and inactivation of caspase-3 (Balsara and Ploplis, 2008). Navitoclax is a BH3 mimetic target and induces senescent cell apoptosis via inhibition of anti-apoptotic Bcl-2 proteins allowing MOMP and cytochrome-C release.

With regards to the heart navitoclax (ABT-263) and the combination of senolytic compounds, dasatinib and quercetin (D&Q) have been investigated. Navitoclax is a Bcl-2 homology 3 domain (BH3) mimetic target and induces senescent cell apoptosis via inhibition of anti-apoptotic proteins Bcl-2, Bcl-XL and Bcl-W (Zhu et al., 2015). Individually dasatinib and quercetin were demonstrated to have a moderate senolytic activity, but in combination, D&Q treatment is an effective senolytic (Zhu et al., 2015). Dasatinib promotes apoptosis via the inhibition of ephrins, which regulate a pro-survival network that includes BCL-xL, PI3K, p21^Cip^, PAI1 and PAI2 (Zhu et al., 2015). Quercetin is a natural flavonol and an inhibitor of multiple pro-survival proteins including PAIs and PI3K (Olave et al., 2010) which when inhibited reduce Bcl-W expression (Bruning, 2013; Olave et al., 2010) (Fig. 2).

## 3 Senescence, heart failure and heart transplantation

### 3.1 Promises

Clinical heart failure (HF) represents a worldwide problem with an estimated 6.2 million adults in the United States currently suffering from HF, and 90 % of these individuals are over 60 years old (Benjamin et al., 2019). During ageing, the heart can undergo structural, biochemical and biomechanical changes including increased arterial stiffening, increased myocardial stiffness (which can be independent of hypertension), increased interstitial fibrosis and CM hypertrophy, decreased diastolic myocardial relaxation, increased LV mass, decreased peak contractility, reduced myocardial and vascular responsiveness to β-adrenergic stimulation, chronic sterile inflammation, and decreased mitochondrial response to increased demand for adenosine triphosphate (ATP) production (Lazzarini et al., 2013).

In particular, ageing is associated with HF characterised by diastolic LV dysfunction with normal LV function (Borlaug and Paulus, 2011), termed HF with preserved ejection fraction (HFpEF). Over 50 % of hospitalized HF patients exhibit HFpEF and the prevalence is growing with an ageing population (Benjamin et al., 2019). HFpEF is expected to increase at a rate of over 10 % per decade (Borlaug and Paulus, 2011).

With rising age, senescence accumulates throughout the myocardium, reflected by an increase in senescent CMs, endothelial, smooth muscle cells, cardiac fibroblasts and cardiac progenitor cells (Anderson et al., 2019; Lewis-McDougall et al., 2019; Rivard et al., 1999; Walaszczyk et al., 2019). In preclinical models of natural ageing, it has been demonstrated that senescence contributes to the pathophysiology of age-related remodelling and cardiac dysfunction. Administration of the senolytic navitoclax to aged-24 month-old mice (two 1-week cyclic doses over a month), reduces CM senescence, attenuates components of the CM SASP, reduces both CM hypertrophy and interstitial fibrosis and reduces LV mass all of which are characteristics of age-associated myocardial remodelling and HFpEF (Anderson et al., 2019; Walaszczyk et al., 2019). Functionally, navitoclax treatment rescues the age-associated decline in diastolic function but does not affect systolic function (Walaszczyk et al., 2019).

Zhu et al have also reported that treatment of aged (24-month-old) mice with a single dose of a combination of D&Q (D: 5 mg kg^−1^ body weight and Q: 50 mg kg^−1^) results in a significant improvement in ejection fraction (EF), a measurement of systolic function. However, no alterations in cardiac mass were observed. Based on the senolytic activity of D&Q on HUVECs in vivo, and observations that in an *ex vivo* assay senolytic treatment improves the vascular relaxation of carotid arteries, Zhu et al. suggest that the clearance of senescent endothelial cells underlies the observed benefits, although no quantification of senescent cell numbers within the heart was performed. Therefore, differences between the outcomes of these studies may reflect the potential cell-type specificity of these two senolytic treatments. Furthermore, differences in the dosing regime and methods of cardiac functional assessment (MRI vs echocardiography), may also have contributed.

The suggestion that CM senescence is causal to age-associated remodelling and HF has long been contentious. As discussed previously, the TERC**^−/−^** mice model has increased our understanding of how myocardial senescence could contribute to heart ageing, however, the clinical relevance of this model is questionable. The degree of telomere shortening in late-generation mice far exceeds the shortening experienced during normal ageing (Rudolph et al., 1999) particularly given the extremely low levels of CM turnover that occurs in the heart during homeostasis (Bergmann et al., 2015; Richardson et al., 2015a). Thus, the CM genome is unlikely to be subjected to excessive end-replication-associated telomere shortening, which is in line with recent studies which demonstrate that whilst shortened leucocyte telomere Length (LTL) is associated with ageing clinically, CM telomere length appears unaltered (Sharifi-Sanjani et al., 2016) and whilst telomerase is expressed in the murine heart no differences in telomerase activity are observed during ageing (Richardson et al., 2012).

**Fig. 3:**
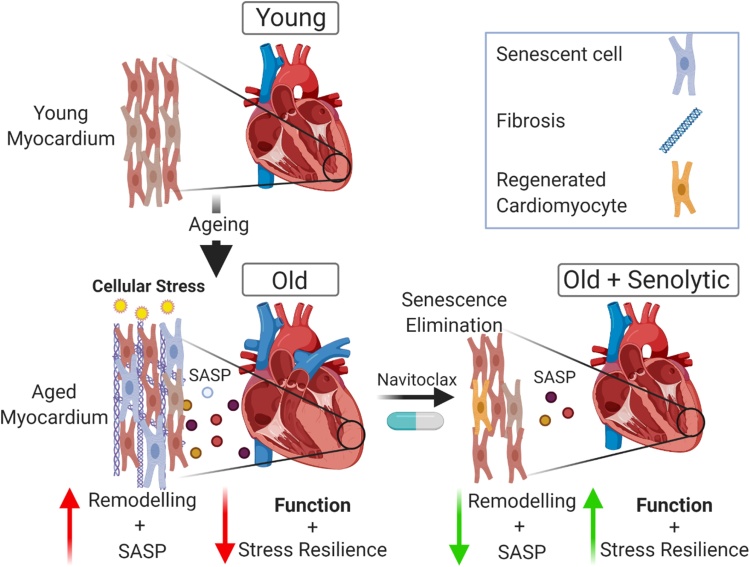
Cardiomyocyte senescence drives age-related cardiac remodelling and dysfunction. During ageing cellular stress including that caused by mitochondrial dysfunction leads to increased oxidative stress and induces TAF in the CM population. Senescent CMs produce an atypical SASP which induces myocardial remodelling. Treatment of mice with a senolytic reduces myocardial senescence, the associated SASP, attenuates remodelling, improves diastolic function and resistance to stress. Elimination of senescence also induces a regenerative response.

It is now demonstrated that CM senescence can be induced as a result of the accumulation of telomere-associated DNA damage foci (TAF) caused by mitochondrial dysfunction and increased oxidative stress, which occurs in the CM population during ageing (Anderson et al., 2019) independent of cellular replication. The senescent CM phenotype is associated with increased hypertrophy and the production of a functional SASP which can induce characteristics of myocardial remodelling in vitro, inhibiting cell proliferation and stimulating myofibroblast differentiation of fibroblasts and causing hypertrophy in healthy neonatal CMs (Fig. 3) (Anderson et al., 2019).

Transcriptomic studies comparing enriched populations of old and young CMs have identified that senescent CMs express an atypical SASP which includes growth and differentiation factor 15 (GDF-15), endothelin 3 (EDN3) and transforming growth factor β2 (TGF-β2), all of which have been associated with cardiac dysfunction, myocardial remodelling and the progression of HF clinically (Anderson et al., 2019). These data may also indicate that CM senescence contributes to age-related HF progression in humans as well as mice as in a metanalysis of 21 original studies, including 20,920 participants, GDF-15 was found to be a strong prognostic indicator of all-cause mortality in HF patients (George et al., 2016). Moreover, elevated serum levels of GDF-15 are associated with enhanced CVD development and progression (Wesseling et al., 2020) and it has been identified as a causal player in adverse myocardial remodelling (Wesseling et al., 2020). Importantly, with regards to age-related myocardial dysfunction, GDF-15 is equally elevated with HFpEF and HF with reduced ejection fraction (Chan et al., 2016), suggesting that GDF-15 may contribute to HF clinically. Together, this data suggests that myocardial senescence contributes to myocardial dysfunction and senescence-induced remodelling (SIR) mediated via the pro-inflammatory SASP, independently of impaired CM renewal as a result of cell cycle exit and progenitor cell dysfunction. Furthermore, the observations that senescent CMs are the largest CMs in vivo, together with the demonstration that treatment with the senomorph rapamycin can reduce senescent burden and hypertrophy but does not influence inflammation suggest that the senescent phenotype is directly associated with cellular hypertrophy (Anderson et al., 2019; Correia-Melo et al., 2019).

That is not to say that myocardial regeneration is unaffected during biological ageing. It has been suggested that the adult heart, including the human heart, contains a rare population of cardiac stem/progenitor cells (CPCs) (Ellison et al., 2013; Eschenhagen et al., 2017; Meeson et al., 2013; Oldershaw et al., 2019; Scalise et al., 2020). Lewis-McDougall et al. have demonstrated that in humans a population of CPCs defined as c-kit^pos^/CD31^neg^CD45^neg^ also accumulate DNA damage, demonstrate significant telomere shortening with age (54–63 years vs 71–79 years) and obtain a senescent phenotype which includes expression of p16^Ink4a^ and SA-β-Gal activity. Senescent CPCs also express a functional SASP which can inhibit the proliferation of healthy CPC populations (Lewis-McDougall et al., 2019). Senescent CPCs exhibit a decline in proliferation and stem cell function, as senescent CPCs lose the potential to enhance regeneration and restore cardiac function following transplantation into infarcted murine hearts (Lewis-McDougall et al., 2019). Supporting the role of SIR in ageing, Lewis-McDougall also demonstrate that D&Q treatment of aged mice attenuates fibrosis and CM hypertrophy in addition to promoting CM turnover and CPC proliferation. While debate remains regarding the extent to which CPCs contribute to myocardial homeostasis, senescence induced CPC dysfunction may contribute to the observed decline in CM turnover that occurs with increasing age (Bergmann et al., 2015; Richardson et al., 2015a).

CPCs are not the only source of CM turnover and renewal; surprisingly, given the heart’s limited regenerative potential, a rare population of CMs retain some capacity for proliferation (Malliaras et al., 2013; Senyo et al., 2013). Elimination of senescent CMs in aged mice stimulates CM proliferation (Anderson et al., 2019), as indicated by the appearance of a population of small mononucleated CMs and an increase in the number of CMs expressing Ki67 and the cytokinesis marker aurora B kinase (Anderson et al., 2019). Together, these studies suggest that following the elimination of senescent CMs, through treatment with navitoclax or D&Q, the myocardium responds with compensatory CM regeneration.

These data could also explain the failure of pre-clinical cellular or regenerative therapies to translate clinically (Lewis-McDougall et al., 2019). Preclinical studies have focused on the use of young healthy animals (Hsieh et al., 2007; Malliaras et al., 2013) whereas CVDs increase in prevalence with age (Yazdanyar and Newman, 2009). Therefore, the majority of patients receiving cellular therapies will have already accumulated myocardial senescence (Anderson et al., 2019; McHugh and Gil, 2018), which would thereby create an unfavourable environment via the SASP (Lewis-McDougall et al., 2019). Senolytic treatment of cells prior to transplantation/engraftment or the host tissues before transplantation may therefore improve the outcomes of such regenerative cellular therapies. A concept supported by the observations that treatment of cultures of senescence CPCs with the senolytic cocktail D&Q reduces senescent CPC numbers and reduces SASP expression in vitro. Furthermore, these data highlight the importance of using aged models for these types of preclinical studies.

Due to the lack of successful regenerative therapies, the only effective treatment for patients with end-stage HF remains transplantation. As a result of the ageing population and the accompanying increase in those suffering from HF, we face the increasing worldwide problem that there are insufficient donor hearts to meet the demand for transplantation. The association between cardiac dysfunction and ageing has led to recommendations that only young hearts should be used for transplants. As such, 70 % of transplants are from donors younger than 49 years of age and hearts from older donors, comprising >65 % of those available, are discarded solely based on chronological age due to fears of increased risk of primary graft dysfunction (PGD). While in general hearts from older donors have an increased susceptibility to PGD (Singh et al., 2019) the reasons for this are not clear, although, biological alterations in aged CMs and a tendency toward reduced cardiac function in older hearts are thought to contribute (Lietz et al., 2004). It is currently speculated that senescence may contribute to donor age-associated increase in allograft dysfunction. Clinically, elevated expression of senescence markers including p16^Ink4a^ in pre-transplant kidney biopsies correlates with reduced organ function following transplant (Koppelstaetter et al., 2008; McGlynn et al., 2009). Furthermore, and highlighting a potential use of senescence as a prognostic biomarker of organ transplant, the use of donor organ p16^Ink4a^ expression as an indicator of biological age, in combination with chronological donor age is better at predicting renal allograft dysfunction than chronological age alone (McGlynn et al., 2009). Further suggesting that senescence is causal to allograft dysfunction rather than being a passive bystander, recipient mice receiving life-supporting kidney transplants from donor mice in which senescence is inhibited (p16^Ink4a^ knockout), survive significantly better than those transplanted with wild-type kidneys (Braun et al., 2012). With regards to the heart, given that senescence accumulation drives age-related myocardial dysfunction and impacts on resilience to stress in vivo (Baker et al., 2016), including ischaemia (Walaszczyk et al., 2019) and ischaemia reperfusion (IR) (Dookun et al., 2020a), senescence accumulation may predispose older transplanted hearts to PGD (Braun et al., 2012). Indeed, preclinical studies using senotherapies have provided a direct link between myocardial senescence and transplant outcome. Iske et al demonstrated that hearts from aged donor animals which contain high levels of myocardial senescence produce a heightened inflammatory response following ischaemia reperfusion injury (IRI) and demonstrate decreased survival subsequent to heterotopic transplant compared to hearts from young (non-senescent) animals (Iske et al., 2020). Elimination of myocardial senescence by providing the donor animal D&Q prior to heart transplant increases allograft survival and attenuates the IRI inflammatory response (Iske et al., 2020). Interestingly, senescent cell-derived circulating cell-free mitochondrial DNA (cf-mtDNA) contributes to the stimulation of host immunity post-transplant, impacting allograft survival (Iske et al., 2020) and plasma levels of cf-mtDNA correlate with myocardial senescence and are a prognostic indicator of PGD (Iske et al., 2020). This, and the observation that older human organ donors have increased circulating cf-mtDNA compared to young donors (Iske et al., 2020), suggests that circulating cf-mtDNA may be a prognostic biomarker for myocardial senescence clinically. These studies also suggest that senolytics could be used to improve the survival of both the organ and recipient post-transplant. For solid organ transplantation, several windows of therapeutic targeting exist, including treatment of the donor, treatment of the recipient, and treatment of the graft itself.

### 3.2 Potential pitfalls

An important question from these studies is whether the loss of senescent CMs caused by senotherapy may be detrimental to the heart. CM loss combined with the limited regenerative potential of the heart underlies the pathophysiology of several cardiomyopathies and the subsequent progression to HF. For example, ischaemic cardiomyopathy (ICM), the most common cause of dilated cardiomyopathy occurs following MI as a result of ischaemic damage to the myocardium leading to extensive CM death and is characterised by extensive maladaptive myocardial remodelling and myocardial dysfunction (Hausenloy and Yellon, 2013). It is therefore perhaps surprising that elimination of senescence CMs in aged mice improves cardiac function and attenuates remodelling. It has been suggested that a lack of regenerative potential following MI could be a result of the ischaemic nature of the disease as even highly regenerative organs such as the liver fail to regenerate in the absence of a patent blood supply (Selzner et al., 1999).

Indeed, it has been previously demonstrated that following acute non-ischaemic myocardial damage, with a single high dose of isoproterenol, the murine heart has a robust cardiac regenerative potential with functional recovery occurring in as little as 28 days (Ellison et al., 2007; Ellison et al., 2013). In contrast to the LAD-Ligation model of MI, which results in a severe and segmental loss of approximately 20–30 % of LV CMs (De Villiers and Riley, 2020), high dose isoproterenol causes diffuse tissue damage that more recapitulates normal muscle wear-and-tear and death in only 8–10 % of apical LV CMs (Ellison et al., 2007, 2013). Therefore, with a diffuse CM death in the absence of an ischaemic insult and with the presence of a patent blood supply, CM regeneration may be sufficient to maintain tissue integrity and function, explaining the observed cardio-beneficial effects of senotherapies in aged mice (Anderson et al., 2019; Lewis-McDougall et al., 2019; Walaszczyk et al., 2019).

In future, it is possible that a combination of senolytic and regenerative cellular therapies could be used to maintain CM number if the human endogenous regenerative potential is insufficient to maintain myocardial muscle function. Interestingly, CM renewal and regeneration can be stimulated by exercise. In both healthy adult mice and adult mice subjected to an experimental model of MI, 8 weeks of monitored voluntary wheel running increased new CM formation (Vujic et al., 2018), suggesting that co-prescription of both pharmacological and non- pharmacological interventions in patients undergoing senolytic treatment may be an alternative strategy to maintain CM number and myocardial function. However, the capacity of the human and murine heart to regenerate may be considerably different.

Following senescence cell clearance, the aged mouse heart not only responds with CM regeneration but also CM karyokinesis in the absence of CM division (Anderson et al., 2018). This process results in bi- or multi-nucleated CMs and is associated with the CM hypertrophy that occurs during adaptive and ultimately maladaptive remodelling (Ahuja et al., 2007; Richardson, 2016). Whilst senescence elimination reduces hypertrophy and improves function at 5 weeks post-treatment, studies are yet to investigate how the heart responds to senolytic treatment in the longer term. It is possible that at later time points ongoing karyokinesis could be detrimental to cardiac function. It remains to be seen how the human heart will adapt following senescent CM apoptosis and what the longer-term outcomes of senolytics will be both for the treatment of HF and in the context of transplantation.

## 4 Myocardial infarction

### 4.1 Promises

Ageing is associated with an increased prevalence of coronary heart disease, the leading cause of death and disability in developed countries (Roger, 2007). The mean age of patients receiving intervention for MI is approximately 65 years old (Alabas et al., 2019) and age is the most important predictor of outcome following MI, with older individuals demonstrating reduced survival and a higher prevalence of HF, compared to younger individuals (North and Sinclair, 2012). MI is characterised by a sustained period of myocardial ischaemia, which results in CM death and cellular necrosis throughout the myocardium with subsequent adaptive, and then maladaptive, myocardial remodelling which is detrimental to cardiac function. In most cases, a MI arises due to the rupture or breakdown of a coronary atherosclerotic plaque that triggers thrombus formation, ultimately leading to blockage of the coronary artery and thus cessation of blood flow distal to the occlusion (Montecucco et al., 2016; Thygesen et al., 2012). Comparable to the clinical situation, aged mice demonstrate increased mortality and poorer outcomes in terms of cardiac function following MI (Boyle et al., 2013; Walaszczyk et al., 2019). However, aged mice treated with navitoclax prior to permanent ligation of the left anterior descending artery (LAD), a model of MI, demonstrate reduced myocardial senescence and an attenuated myocardial SASP which results in increased survival and improved functional cardiac outcomes (Walaszczyk et al., 2019). These data suggest that senescence which accumulates during natural ageing reduces the hearts resilience to cardiac stress, an observation supported by others (Baker et al., 2016).

The most serious manifestation of coronary heart disease is ST-segment elevation MI (STEMI), which is caused by complete occlusion of a coronary artery (Lonborg, 2015). The most effective treatment is timely reperfusion of the myocardium via primary percutaneous coronary intervention (PPCI) (Lonborg, 2015). Despite the advantageous effects of PPCI and other revascularisation methods, the resulting reperfusion after an ischaemic event can detrimentally impact and exacerbate myocardial dysfunction via IRI (Hausenloy and Yellon, 2013; Lonborg, 2015). Numerous clinical conditions besides MI have been linked to the occurrence of IRI. These include, but are not limited to, stroke, organ transplantation and peripheral vascular disease (Kalogeris et al., 2012; Widgerow, 2014). Regardless of its systemic prevalence, other than the initial (inevitable) ischaemic insult, the exact mechanisms behind IRI remain unclear so it cannot be exploited therapeutically. This underpins the need to identify additional therapies to either prevent or ameliorate myocardial IRI, which has been described as a neglected therapeutic target (Hausenloy and Yellon, 2013). The current understanding of the underlying pathophysiology is highly complex and multifactorial (Halladin, 2015; Hausenloy and Yellon, 2013; Neri et al., 2017). However, a key component of IRI is the increased generation of ROS (Chouchani et al., 2014) and increased cellular stress which has recently been shown to cause DNA damage in the form of TAF and induce myocardial senescence (Dookun et al., 2020b).

**Fig. 4:**
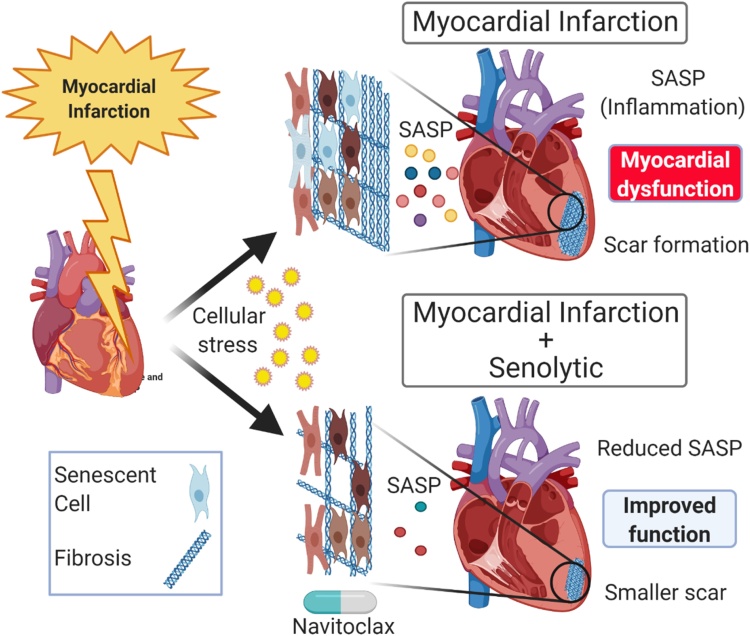
Senescence contributes to myocardial remodelling and cardiac dysfunction post-cardiac ischaemia reperfusion. The cellular stress caused by IR induces senescence in both CMs and interstitial cells. Senescent cells produce a SASP that contributes to fibrosis (scar formation) and inhibits revascularisation. Treatment of mice with the senolytic navitoclax after IR attenuates myocardial inflammation, reduces scar size, and increases revascularisation, which together improves cardiac systolic function.

Using a mouse model of IRI, in which LAD-ligation is performed for 60 min. followed by the return of the blood supply allowing reperfusion of the myocardium, Dookun et al. demonstrated that ischaemia reperfusion (IR) induces senescence in both CMs and interstitial cell populations (Dookun et al., 2020b). As with natural ageing, this IR-induced senescence is detrimental to myocardial function and promotes myocardial remodelling. Treatment with navitoclax, at a clinically feasible timepoint of 4 days post-IR, reduced the senescence cell burden, improved LV function, attenuated remodelling, increased myocardial vascularization, and decreased scar size (Dookun et al., 2020b) (Fig. 4). Suggesting that these beneficial effects may be a result of attenuation of the SASP, proteomic analysis identified that protein networks that were significantly increased in LV myocardium post-IR and related to inflammation and fibrosis, were significantly reduced following senescent cell reduction (Dookun et al., 2020b). Moreover, senolytic treatment also reduces the expression of proinflammatory, profibrotic and anti-angiogenic cytokines, including C-C motif chemokine ligand 22 (CCL22), interleukin 6 (IL‐6), interleukin 11 (IL‐11), interferon gamma-induced protein 10 (IP‐10), eotaxin, and fractalkine (Dookun et al., 2020b). These cytokines are associated with nuclear factor‐κB (NF‐κB) signalling (Bhavsar et al., 2008; Bitko et al., 1997; Hein et al., 1997; Nakayama et al., 2004; Shultz et al., 2007; Son et al., 2008) which is a well-established modulator of the acute inflammatory phase following IR (Boag et al., 2015). Excessive NF‐κB signalling is linked to increased maladaptive remodelling, increased scar size and poorer functional outcomes post-IR (Campanella et al., 2010; Hilfiker-Kleiner et al., 2010; Obana et al., 2010; Prabhu and Frangogiannis, 2016) and increased serum levels of potential SASP cytokines including IL-6, fractalkine and IL-11 correlate with a poorer outcome to MI and HF clinically (Boag et al., 2015; Groot et al., 2019; Spray et al., 2021; Ye et al., 2019). Together these findings suggest that senescence accumulation and SASP may contribute to remodelling post-MI in humans and that senotherapies may provide a strategy to target the cellular source of multiple cytokines and chemokines, known to be detrimental to recovery clinically. Thus, cellular senescence represents a potential novel therapeutic avenue to improve patient outcomes following cardiac IR.

### 4.2 Potential pitfalls

While these studies are encouraging and highlight the potential for the clinical use of senolytics, they do not fully replicate the clinical situation, in which MI occurs primarily in older patients. One concern may be that with age there is already a degree of myocardial senescence prior to the infarct. In older individuals senolytic treatment subsequent to IR may therefore induce apoptosis in a larger senescent cell population and this together with an age-associated decline in regenerative potential (Bergmann et al., 2015; Richardson et al., 2015a) due to an accumulation of stem cell dysfunction (Lewis-McDougall et al., 2019) may lead to detrimental outcomes. Before senolytics can be tested as a clinical therapeutic post-IR, studies using mice of a clinically relevant age are required to investigate the effect of the elimination of senescent cells induced by IR against a background of age- accumulated senescence. As with age-associated cardiac remodelling, long term survival studies will also be necessary to understand if and how the short-term benefits from senolytic therapy which are observed despite the loss of CMs, are maintained in the medium-to longer-term and translate to improved survival.

The observations that senolytics can attenuate inflammation following IR may be considered a beneficial effect, however following IR the cytokine response has pleiotropic effects and although increased inflammation can promote adverse remodelling it is also integral to resolving tissue injury (Hanna and Frangogiannis, 2019). The reparative phase of the response to heart injury is characterized by the secretion of TGF‐β1, an anti‐inflammatory and profibrotic cytokine responsible for the switch from the pro‐inflammatory phase to the resolution phase of cardiac healing and driving formation of the fibrotic scar (Hanna and Frangogiannis, 2019). Early neutralization of TGF‐β signalling at 24 h post‐MI is detrimental, increasing both cardiac dysfunction and mortality, whereas late disruption of TGF‐β signalling is protective for fibrosis and hypertrophic remodelling (Ikeuchi et al., 2004). Subsequent to IR in mice, myocardial TGF‐β expression correlates with the levels of myocardial senescence, being increased following IR and reduced upon senescent cell clearance, suggesting TGF-β expression post-IR is at least in part a component of the SASP (Dookun et al., 2020b). As such, the different outcomes caused by either late or early TGF-β inhibition highlight the potential importance of the correct timing of senolytic treatment. Furthermore, some SASP components are known to be beneficial to recovery following IR clinically, for example, increased expression of the cytokine IP-10, which was reduced by senescence elimination in preclinical models (Dookun et al., 2020b), at 15 min post-IR correlates with higher systolic function at 12-weeks post-IR in patients, indicating that expression of IP-10 in the acute phase following MI confers cardioprotection (Shmeleva et al., 2015). Senolytic treatment regimens will therefore need to take into consideration the temporal dynamics and composition of the inflammatory and immune response.

Systemic attenuation of senescence via knock-out of p53 or p53 and p16^Ink4a^ results in exaggerated fibrosis following myocardial insult, suggesting that senescence induction represses myocardial fibrosis (Meyer et al., 2016; Zhu et al., 2013). Mice lacking p53 displayed decreased myocardial senescence, an increase in fibrotic areas and an increase in the expression of collagen I and collagen II following MI when compared to wild type controls (Zhu et al., 2013). Similarly, mice lacking p53 and p16^Ink4a^ subjected to transverse aortic constriction (TAC), a commonly used model of pressure overload-induced myocardial remodelling and fibrosis (Richards et al., 2019), displayed more extensive fibrosis and reduced cardiac function (Meyer et al., 2016). Conversely, when senescence was induced prematurely via adeno-associated virus gene transfer of cellular communication network factor 1, an inducer of premature senescence (Jun and Lau, 2010) myocardial perivascular fibrosis was reduced by approximately 50 % following TAC (Meyer et al., 2016).

Whilst the observed reduction in scar size following navitoclax treatment initially appears contradictory with these studies, it is important to note that navitoclax does not inhibit senescence but rather induces apoptosis once senescence is achieved (Zhu et al., 2015). Therefore, these data are entirely compatible. Indeed following MI mice lacking expression of p53 also developed a heightened inflammatory response characterised by IL-1, chemokine ligand 1 (CXCL1), CXCL2, monocyte chemoattractant protein 1 (MCP-1), IL-6, granulocyte chemotactic protein 2 (GCP-2) and macrophage colony-stimulating factor (M-CSF) (Zhu et al., 2013), known inducers of fibrosis (Schafer et al., 2018; Zhu et al., 2013). As such, while in the short-term senescence and the SASP may limit fibrosis, an accumulation of senescent fibroblasts post-MI might also contribute to chronic inflammation exacerbating ongoing cardiac collagen deposition and fibrosis formation.

It should also be highlighted that myocardial fibrosis and scar formation are not merely maladaptive processes, but rather are compensatory mechanisms initially required to maintain ventricular myocardial structure and thereby retain function and structural integrity following extensive CM loss. While the findings of studies showing that following MI senolytic treatment reduced fibrosis (Dookun et al., 2020b), at first appear entirely positive, reduced fibrosis could also have detrimental consequences. During the first days following infarction the infarct zone is associated with CM necrosis and the recruitment of immune cells which remove necrotic CMs and cellular debris, so the infarct is at its mechanically weakest (Richardson et al., 2015b). Prior to the development of reperfusion therapies a common catastrophic complication of MI was cardiac rupture, with an incidence between 7–20% in STEMI patients between the 1970s and 1990s (Lu et al., 2020). Now that reperfusion therapy is common practice the incidence has fallen to approximately 1% (Lu et al., 2020). While the precise aetiology of cardiac rupture is unclear, failure to produce a scar with the required structural integrity and extensive CM apoptosis contribute to reduced tensile strength of the infarcted myocardium (Gao et al., 2012). As such, senolytic elimination of senescent myofibroblasts and senescent CMs in the border of the infarct could theoretically increase susceptibility to rupture. Although, it should be highlighted excessive inflammation and increased NF-κB, and MMP signalling which is attenuated by serotherapy in mice (Dookun et al., 2020b), are associated with increased prevalence of cardiac rupture (Gao et al., 2012).

Senescence plays a complex role in coordinating inflammation, fibrosis, tissue remodelling, and wound healing following MI, and senescence and the SASP may have some beneficial consequences following myocardial injury which could be impacted by senolytic treatment. Further studies are required to determine how senolytic therapies can be best implemented to gain the most therapeutic benefit whilst avoiding deleterious short- and long-term outcomes. This will require studies to be undertaken in appropriately aged animals, investigating not only the timing of therapeutic intervention but also the long-term outcomes.

## 5 Atherosclerosis and the potential to treat thoracic aortic aneurysms and calcific aortic valve disease

### 5.1 Promises

Atherosclerosis is a leading cause of vascular disease worldwide, initiated by endothelial injury or accumulation of low-density lipoproteins (LDLs) within the arterial wall which leads to the development of lipid and protein-filled ‘plaques’ triggering both the innate and adaptive immune responses (Moriya, 2019). Inflammation stimulates necrotic core enlargement, extracellular matrix degeneration and cap thinning, erosion, calcification, and intra-plaque angiogenesis (Stojanović et al., 2020). As atherosclerosis progresses plaques become unstable and eventually thrombogenic. Rupture causes vessel obstruction leading to severe ischaemic injuries including MI and stroke (Moriya, 2019). Using low-density lipoprotein receptor-deficient (*Ldlr***^−/−^**) mice on a high-fat diet, a model of atherogenesis, senescence was identified in the plaque-rich aortic arches as indicated by elevated transcript levels of the cyclin-dependent kinase inhibitor p16^Ink4a^ and typical SASP proteins including MMP-3, MMP-13 and the inflammatory cytokines IL-1α and TNFα (Childs et al., 2016). Atherosclerosis is associated with senescence of multiple different cell types including endothelial cells, vascular smooth muscle cells (VSMC), monocytes, foam cells and T-cells (Song et al., 2020) all of which contribute to increased inflammation. Endothelial cell senescence contributes to the progression of atherosclerosis through increased and chronic adhesion to monocytes, due to demethylation of the CD44 promoter and increased expression of CD44 (Lowe and Raj, 2014). Moreover, subpopulations of monocytes also become senescent with age and can also promote vascular inflammation and thereby atherogenesis and atherosclerosis. Senescent monocytes display elevated endothelial cell adhesion, an activated phenotype, demonstrate increased cytokine release and an increased ability to induce lymphocyte proliferation (Merino et al., 2011).

**Fig. 5:**
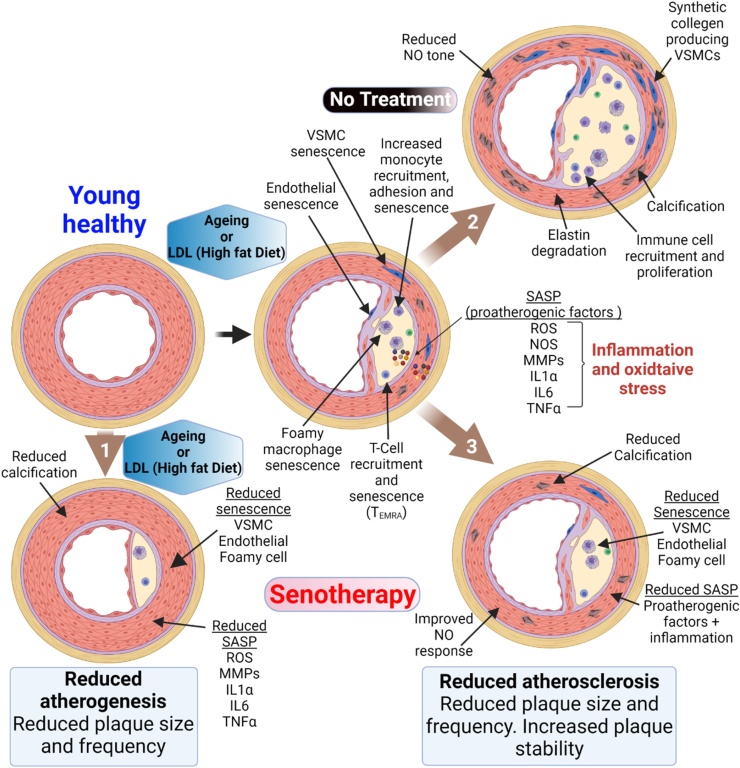
Senescence and senotherapy in atherosclerosis. Studies using senolytics or transgenic models that enable senescent cell elimination have demonstrated that senescence and the SASP contribute to atherogenesis and atherosclerosis. Young healthy arteries have low levels of senescence. During ageing or as a result of increased LDL, senescence accumulates in multiple cell populations including endothelial cells, VSMCs, T-cells, monocytes, and foamy cells. In preclinical models, if senescent cells are eliminated before the onset of atherogenesis there is a reduction in senescence cell burden, reduced expression of the proatherogenic SASP, reduced calcification and an associated reduction in plaque size and number (**arrow 1**). If ageing or a high-fat diet continues in the absence of senescent cell elimination, increased senescent burden and SASP contributes to increased inflammation and a proatherogenic environment. Senescence of endothelial and VSMCs contribute to loss of NO tone and the differentiation of the synthetic VSMC phenotype. The expression of the proatherogenic and proinflammatory SASP promotes plaque development and instability by provoking VSMC hyperplasia, medial thickening, collagen overproduction, synthesis of elastin-degrading proteins (metalloproteases), calcification and further immune cell recruitment. Inflammation and the SASP may further induce senescence in multiple cell types enhancing plaque development and instability (Arrow 2). Elimination of senescent cells following atherogenesis reduces the proatherogenic SASP, improves vessel tone, reduces calcification and decreases plaque size and frequency while decreasing the expression of indicators of plaque instability including elastic fibre degradation, fibrous cap thinning and metalloprotease production. (Arrow 3).

Despite the observations that senolytics have the potential to improve outcome following MI (Dookun et al., 2020b), prevention is better than cure. Recently two studies have provided tantalising evidence that senotherapies prevent or slow the progression of atherosclerosis (Childs et al., 2016; Roos et al., 2016). Treatment of atherosclerotic *Ldlr***^−/−^** mice with navitoclax, after senescence is established, reduces senescent cell numbers, diminishes plaque burden, plaque number and the average plaque size (Childs et al., 2016). This is also associated with a reduction in factors implicated in plaque formation, such as MCP1, IL-1α, TNFα and the leukocyte receptor vascular cell adhesion protein 1 (Childs et al., 2016). The potential for senotherapies to abate atherosclerosis is also provided by the studies of Roos et al., using *ApoE****−/−*** mice, an alternative model of atherogenesis, treatment with D&Q reduces senescence cell burden and plaque calcification but does not influence plaque size (Roos et al., 2016). Importantly, and suggesting that the above observation was indeed a result of senescence elimination and not due to secondary effects of the senolytic compounds, similar beneficial effects have been reported when senescence was eliminated using transgenic mouse models (Fig. 5).

Shorter leukocyte telomere length (LTL) is linked with CVD clinically. Short LTL is predictive of higher overall mortality in over 60-year-olds, an increased risk of death from heart disease, severe HF, atherosclerosis and the development of ischaemic heart disease and may be predictive for ventricular arrhythmias in patients with ischaemic heart disease, as reviewed previously (Fernández-Alvira et al., 2016; Hoffmann et al., 2021; Valdes et al., 2005). Furthermore, short LTL is also associated with cardiovascular risk factors such as hypertension, stress, smoking, or obesity (Valdes et al., 2005). However, LTL is not a significant independent determinant of subclinical atherosclerosis in middle-aged populations (Fernández-Alvira et al., 2016; Valdes et al., 2005). Therefore, debate remains as to whether decreased LTL contributes to these CVDs or LTL shortening occurs as a result of the chronic inflammation and oxidative stress associated with these diseases (Hoffmann and Spyridopoulos, 2011). For example, activated T-cells express telomerase to prevent replicative senescence (Weng et al., 1996). Oxidative stress can suppress T-cell telomerase expression (Richardson et al., 2018) and as such chronic oxidative stress within an atherosclerotic plaque could accelerate telomere attrition instigating T-cell senescence. Interestingly, although oxidative stress inhibits telomerase activity in T_reg_ cells, which have anti-atherosclerotic functions, it does not affect their proliferation (Richardson et al., 2018). Consequently, proliferation in the absence of telomerase expression could accelerate T**_reg_** senescence promoting atherosclerosis. Furthermore, statins, one of the most potent drugs in delaying age-related inflammatory changes in the arterial vessel wall and that slow the progression of atherosclerosis have been shown to stimulate telomerase activity (Bennaceur et al., 2014). While telomerase has enzymatic functions beyond those of telomere maintenance (Hoffmann et al., 2021), supporting the hypothesis that oxidative stress mediates T-cell telomere shortening inducing senescence which in turn promotes atherogenesis are the observations that CD8**^+^** T-cells become functionally senescent with age (Callender et al., 2018). T-cell senescence is implied by proliferative arrest and the increased production and secretion of inflammatory mediators characteristic of a SASP (Callender et al., 2018), and once senescent, cytotoxic terminally differentiated CD8**^+^** T cells (TEMRA) are pro-atherosclerotic and an independent predictor of all-cause mortality in the elderly (Martin-Ruiz et al., 2020). While it remains to be seen if senolytic elimination of senescent immune cells can protect against CVDs, it has been demonstrated that navitoclax treatment of aged mice decreases CD8**^+^** effector memory cells and increases the naïve CD8**^+^** T-cell population, a cellular composition associated with young animals and indicating a reduction of immunosenescence (Martin-Ruiz et al., 2020). Whether this reduction in immunosenescence and the associated reduction in the cytotoxic, proinflammatory phenotype contributed to the anti-atherosclerotic effects observed by Roos et al (Roos et al., 2016) and Childs et al. (Childs et al., 2016) remain to be seen. However, the link between inflammation and CVD is well established as reviewed (Liu et al., 2020) and it has been identified that inhibition of excess inflammation can prevent CVD. In the recent CANTOS trial canakinumab, which targets IL-1β inhibiting inflammation, reduced cardiovascular events compared with the placebo arm (Ridker et al., 2017). Furthermore, the link between systemic inflammation and CVD is highlighted by parabiotic experiments in which the hearts of young animals acquired HFpEF-like features when exposed to blood from old animals and vice versa (Loffredo et al., 2013).

Thoracic aortic aneurysms (TAA) are an important cause of cardiovascular mortality with a reported incidence of more than 10 per 100,000 in the general population (Clouse et al., 1998). TAA is associated with a life-threatening risk of aortic rupture or dissection and current medical management involves regular imaging to determine the rate of progression, aggressive blood pressure management and surgical intervention, typically indicated by the size of the aneurysm, associated valvular disease or the occurrence of dissection/rupture (Hiratzka et al., 2010). Surgery of the thoracic aorta is regarded as high risk, so we should have a full understanding of the pathophysiology of this condition to facilitate prediction of expansion and complications, enabling surgery to be undertaken more frequently in the elective setting and ideally develop more targeted therapies to slow down or even prevent aneurysm formation altogether.

While the development of TAAs is likely multimodal, an area of increasing interest is the association of bicuspid aortic valve (BAV) with TAA. As opposed to a typical tricuspid aortic valve (TAV), BAV is a common abnormality that occurs in 1–2 % of the population (Tadros et al., 2009b). BAV is frequently associated with dilation of the ascending thoracic aorta and a significantly increased risk of aortic dissection (Tadros et al., 2009a). There is debate about whether the aneurysmal changes in the aorta with BAV are secondary to haemodynamic changes associated with valvular disease or due to an underlying defect in the wall of the aorta itself. Several features strongly suggest the latter, including the observation of the continued progression of aneurysmal changes despite valve replacement and the finding of TAA with normal aortic valve morphology in first-degree relatives of those with BAV (Biner et al., 2009). Pathologically, TAA is associated with loss of VSMC and degenerative changes in the extracellular matrix, with disruption of the normal elastin pattern (Tadros et al., 2009a). There is also evidence of inflammatory changes, particularly in the adventitial layer (Bonderman et al., 1999). At the molecular level, there is considerable interest in the expression of MMPs and their inhibitors (tissue inhibitors of metalloproteinases - TIMPs). Increased MMP expression is a ubiquitous finding in TAA; whereas there is a minimal expression of these compounds in normal aortic tissue (El-Hamamsy and Yacoub, 2009). Furthermore, and perhaps contributing to these inflammatory changes, a proportion of smooth muscle cells (SMCs) in aneurysmal aortas demonstrate premature senescence (Balint et al., 2019).

Senescence is associated with TAA in patients with both TAV and BAV, however, only aortas of individuals with BAV and not those with TAV contained senescence SMC in the absence of TAA, suggesting both that SMCs in BAV aortas have an increased predisposition to senescence and that SMC senescence contributes to the development of TAAs independently of valve morphology (Balint et al., 2019). SMC senescence is associated with persistent DNA damage in the form of double-stranded DNA breaks and the expression of a collagen-degrading SASP. Aged BAV SMCs express a collagen destructing phenotype characterised by increased expression of interstitial collagenases MMP1 MMP8 and MMP13 and TGF-ß1, and down-regulation of TIMPs1–4 and the collagen α-chains encoding mRNAs COL1A1, COL1A2, and COL3A1. These data indicate that VSMC senescence participates to TAA via the production of collagen destructive SASP, a hypothesis supported by the observations that senescent SMCs in BAV aortas are surrounded by disrupted collagen fibrils and both aneurysmal and non-aneurysmal BAV aortas display reduction in total medial collagen content compared to non-aneurysmal TAV aortas (Balint et al., 2019). While there is no data directly demonstrating that senotherapy could be used to prevent or reverse the formation of TAA or specifically attenuate this degenerative aortic SMC phenotype, data provided by the studies of Childs et al. and Roos et al. indicate that transgenic or senolytic mediated elimination of cellular senescence from the aorta of atherosclerotic mice reduces expression of MMPs including Mmp3, Mmp12 and Mmp13, within aortas (Roos et al., 2016).

These same studies additionally demonstrate that elimination of senescent SMCs from atherosclerotic aortas also reduced both aortic calcification and the expression of osteogenic signalling in the aortas of hypercholesterolemic aged mice (Roos et al., 2016), data which implicates senolytics as potential therapies for calcific aortic valve disease (CAVD) which is also associated with increased aortic senescence (Oh et al., 2021). CAVD affects more than 5.2 million people in the US and the only effective treatment is valve replacement, this now being the second most prevalent cause for heart surgery annually in North America (Lerman et al., 2015), which is not guaranteed to have long-term success (Dutta and Lincoln, 2018). CAVD is an age-related calcifying degenerative disorder of the valve leaflets which is prevalent in the ageing population. Senescent VSMCs are now thought to drive CAVD, as there is an accumulation of senescent VSMC, determined by p16^inka^and p53-positive expression, in the aortic valves of individuals with advanced CAVD (Oh et al., 2021). Furthermore, these senescent VSMCs overexpress proteins that promote osteoblastic transdifferentiation including RUNX-2, alkaline phosphatase type I collagen and BMP-2 which are postulated to promote calcification and fibrosis, and there is a direct correlation between tissue remodelling severity in the aortic valve and the levels of senescent marker expression in the aorta (Burton et al., 2010). Interestingly although CAVD is primarily an age-related disease it can manifest in younger individuals with BAV (Dutta and Lincoln, 2018), providing a further indication that these individuals may have a propensity for senescence or that having a BAV and the associate changes in the haemodynamic of blood flow through the aorta induces cellular stress that promotes senescence in the surrounding tissues.

### 5.2 Potential pitfalls

Death from TAA is due to the catastrophic rupture of the aorta as a result of a failure of tissue integrity (Hiratzka et al., 2010). While TAA and dissection are defined by inflammation and ECM degradation, cell loss also plays a role (Quintana and Taylor, 2019). Specifically, loss through apoptosis or necroptosis of SMCs is a crucial feature of TAA (Quintana and Taylor, 2019). As senescent SMCs would be a major target for senolysis in the treatment of TAA, senotherapies may in fact have the potential to weaken the aorta and promote dissection. There is currently interest in the use of cellular or stem cell therapies for the treatment of TAA (Yamawaki-Ogata et al., 2014), combination therapy using both senolytics and cellular therapies may offer a strategy to circumvent these potential obstacles. Furthermore, if senotherapy is detrimental to TAA, the association between CAVD and TAA, particularly in those with BAV, may lead to barriers to the use of senolytics for the treatment of CAVD. Complications caused by TAA could also limit the value of serotherapy for the treatment of atherosclerosis due to the association of these two diseases.

While the western fed ApoE^−/−^ and *Ldlr***^−/−^** mice models used by Roos et al. and Childs et al develop aortic atherosclerosis, they do not model aortic aneurysm or dissection (Childs et al., 2016; Roos et al., 2016). Administration of angiotensin II to these models has been shown to induce aneurysm (Daugherty et al., 2000; Tanaka et al., 2018) and as such this model may represent a tool to address the above concerns. Interestingly, angiotensin II has also been shown to induce VSMC senescence, leading to vascular Inflammation via a p53/p21^Cip^ dependent mechanism (Kunieda et al., 2006). Data which further implicates premature senescence in the pathophysiology of aortic aneurysm development and progression and provides further weight to the argument that predisposition to senescence in those with BAV may underlie the increased prevalence of TAA in this patient population.

While the elimination of senescent cells from aged mice and models of age-related disease confers benefits, studies have also described beneficial roles for senescent cells (Demaria et al., 2014b). Furthermore, in a recent study, Grosse et al. used a novel transgenic model that allows the detection and elimination of p16^High^ expressing cells (Grosse et al., 2020). These studies demonstrate that p16^High^ expressing cells include cells of the vascular endothelial and macrophage linages and are present in the liver, lung and heart of aged mice (Grosse et al., 2020). Elimination of p16^High^ expressing cells was non-replaceable and was detrimental to health and lifespan contributing to the disruption of the blood-tissue barrier and results in liver fibrosis (Grosse et al., 2020). Furthermore, the authors reported increased perivascular collagen in both the lung and heart. Interestingly, CD31 positive, p16^High^ endothelial cells isolated from this transgenic line had significantly increased acetylated LDL uptake and p16^High^ elimination resulted in a significant increase in plasma oxidized-LDL, a major contributor to atherosclerosis and an inducer of senescence (Oh et al., 2017). Therefore, senotherapies have the potential to promote biological processes associated with atherogenesis. However, importantly D&Q treatment is ineffective at inducing apoptosis in these p16^High^ CD31-positive endothelial cells (Grosse et al., 2020) it remains to be seen how these cells are affected by other senolytics including navitoclax.

## 6 Chemotherapy-induced heart failure

### 6.1 Promises

Improved anti-cancer therapies have increased patient survival, but this has unfortunately led to an increase in patients suffering from complications of chemotherapy. In particular, cardiotoxicity related to chemotherapy is a major healthcare problem and chemotherapy-induced HF can manifest decades after treatment and has a three-fold higher mortality rate than idiopathic dilated cardiomyopathy (Nair and Gongora, 2016). CVD is now considered the leading cause of death in cancer survivors. While chemotherapy has acute cardiotoxicity, it has also been proposed to lead to HF via the accumulation of myocardial senescence (Mitry et al., 2020). Anthracyclines, including the antibiotic doxorubicin (Doxo) a commonly used chemotherapy for both adult and childhood cancers, are associated with late progression cardiomyopathy with 1%–5% of cancer survivors treated with anthracyclines developing HF (Oliveira et al., 2014). Chemotherapy-induced cardiomyopathy is both iatrogenic and predictable with increasing dose (Oliveira et al., 2014). Doxo causes mitochondrial DNA damage, increases oxidative stress in CMs and CPCs results in cellular senescence (Maejima et al., 2008). If Doxo induced myocardial senescence contributes to myocardial dysfunction as a result of SIR, as with ageing, the use of senolytics post-chemotherapy could therefore improve cardiac function via the clearance of senescence and attenuation of myocardial SASP. This hypothesis is supported by the observation that several short- and long-term effects caused by Doxo induced cardiomyopathy including cardiac dysfunction, decreased physical activity and strength are attenuated following transgenic elimination of senescence, using the p16-3MR mouse model (Demaria et al., 2017). Similarly, Poly (ADP-ribose) polymerase 1 (PARP1) inhibitors which are used as maintenance therapy for the treatment of high-grade serous epithelial ovarian cancer (Ledermann et al., 2012) induce human ovarian cell lines to a reversible senescent-like phenotype in vitro, characterised by an inhibition of proliferation via activation of the p21^Cip^ pathway, increased DNA damage, SA-B-gal activity and expression of typical SASP proteins including IL6 and IL8 (Fleury et al., 2019). While this phenotype is transient, senescent-like ovarian cells are sensitive to several previously described senolytics including navitoclax, Fisetin and D&Q (Fleury et al., 2019). In pre-clinical xenograft models of ovarian cancer, combination treatment with PARP1 inhibitors and navitoclax reduces tumour burden compared with PARP1 inhibitors alone (Fleury et al., 2019). Presumably, this was a result of a two-step process, in which PARP inhibition induces a senescent-like phenotype in the tumour cells which sensitises them to senolytic mediated apoptosis. While the use of PARP1 inhibitors is not associated with increased prevalence of HF, perhaps because of the transient nature of the senescent phenotype induced, these studies provide proof of principle data that suggests senolytics have the potential to eliminate chemotherapy-induced senescent myocardial cells.

### 6.2 Potential pitfalls

While originally cellular senescence and therefore chemotherapy-induced senescence was considered as only beneficial in the prevention of cancer progression, it is now clear that the role of senescence as an antitumorigenic mechanism is more complex. As discussed above, in some cases senescence can be reversible and tumour cells that escape senescence have been reported to develop more aggressive phenotypes associated with increased stemness and drug resistance (Triana-Martínez et al., 2020). While senescence is a potent anti-cancer mechanism and the SASP can promote immune cell activity, immune surveillance and tumour clearance, the SASP also has properties that promote malignancy and tumour growth, such as immunosuppression, a stimulus of cell growth and increasing stemness in neighbouring cells. It is also unclear what selective pressure senolytics will place on cancer cells. Treatment could place a selective advantage on cancer cells that lack sensitivity to senolytic compounds or that can evade senescence. For example, cancer cells lacking p53 and p16^Ink4a^ may escape senescence induction and senotherapy could promote clonal expansion of these populations which may also be resistant to chemotherapies that work by inducing cell cycle exit. It remains to be seen if senolytics are a viable strategy to prevent or elevate chemotherapy-induced HF without promoting tumorigenesis.

## 7 Cardiovascular pitfalls impeding clinical translation of preclinical studies

### 7.1 Senolytics and thrombocytopenia

Thrombocytopenia is the dose-limiting toxicity of navitoclax caused by inhibition of Bcl-X_L_ within the platelet population (Schoenwaelder et al., 2011). Although studies have reported thrombocytopenia as a result of navitoclax as an anti-cancer treatment, it caused no clinically relevant bleeding events and thrombocytopenia was fully reversed upon cessation of drug use (Rudin et al., 2012; Zhang et al., 2007). Similarly, studies have shown that >40 % of patients receiving dasatinib therapy for chronic myeloid leukaemia (Quintás-Cardama et al., 2009) present with thrombocytopenia. Furthermore, dasatinib treatment is associated with platelet dysfunction and can induce bleeding, independent of thrombocytopenia (Ozgur Yurttas and Eskazan, 2019). While the mechanism is unclear dasatinib may cause platelet dysfunction via the inhibition of key kinases that play a role in platelet homeostasis (Ozgur Yurttas and Eskazan, 2019).

Thrombocytopenia and platelet dysfunction may result in cardiac-specific complications. Ordinarily, platelets do not adhere to the intact non-activated endothelium, however, in atherogenesis, the inflammation and the production of ROS associated with plaque development activate endothelial cells and platelets allowing adhesion (Badimon et al., 2012). Activated platelets release mediators that stimulate further cell adhesion, survival, and proliferation as well as factors associated with coagulation and proteolysis and secrete proinflammatory cytokines all of which promote plaque development. Following erosion or rupture of an atherosclerotic plaque through coagulation and fibrin production, platelets contribute to thrombus formation. As such, after PPCI STEMI patients are commonly treated with a combination of antiplatelet and anticoagulant drugs to prevent thrombus formation at the site of coronary intervention (Sinkovič and Majal, 2015). Antiplatelet agents, such as aspirin, thienopyridines and glycoprotein-IIb/IIIa receptor blockers are also used as preventatives for ischaemic heart disease. Although platelet inhibition can be therapeutic, care must be taken when using antiplatelet drugs to avoid thrombocytopenia. As well as being associated with gastrointestinal bleeding (Pipilis et al., 2014; Quintana and Taylor, 2019) thrombocytopenic patients with unstable angina or STEMI have an increased risk of experiencing clinically significant ischaemic events including death, nonfatal MI, stroke, and recurrent ischaemia (McClure et al., 1999). The increased risk associated with the use of antiplatelets and anticoagulants on patients with thrombocytopenia has led to fewer PPCIs being performed in this patient cohort. Together this increased risk and the related reduction in intervention linked with thrombocytopenia contributes to a 2-fold increase in 30-day and six-month mortality in those with acute coronary syndromes (Sinkovič and Majal, 2015). Clearly, the platelet apoptosis-inducing activity of navitoclax, the increased risks of cardiac events in patients with thrombocytopenia and the potential interactions between navitoclax and current PPCI therapies will hinder the translational potential of navitoclax clinically for cardiovascular indications.

A derivative of navitoclax, ventoclax (ABT-199) provides a possible alternative to circumvent problems associated with thrombocytopenia. Ventoclax has a higher affinity to Bcl-2 and reduced affinity to Bcl-X_L,_ Bcl-W and MCL-1, and thereby demonstrates decreased platelet toxicity (Souers et al., 2013). The cardiosenolytic potential of ventoclax has not been evaluated, however, treatment is protective against TAC-induced myocardial hypertrophy, an observation that may in part be a result of senolysis. Alternatively, the on-target toxicity of navitoclax has been reduced by converting it into a Bcl-xl proteolysis-targeting chimaera (PROTAC) that spares platelets (He et al., 2020).

### 7.2 Senolytics and cardiotoxicity

There is emerging evidence that suggests dasatinib can result in potentially rare but clinically relevant cardiotoxic effects. 1–10% of patients with chronic myelogenous leukaemia treated daily with dasatinib demonstrate adverse cardiac effects including prolongation of the QT interval, arrhythmia and palpitations (Xu et al., 2009). In rare cases, less than 4%, severe CHF or ventricular dysfunction has also been reported (Brave et al., 2008). The mechanisms underlying dasatinib induced myocardial dysfunction are unknown, although it has been suggested that adversely affected mitochondrial membrane potential and increased apoptosis may contribute (Xu et al., 2009). As such, it is possible that senolysis could contribute to cardiac dysfunction, although this remains to be investigated, however, contradictory to this hypothesis navitoclax has no significant cardiac toxicity (Wilson et al., 2010). Moreover, treatment for chronic myelogenous leukaemia requires an extended daily treatment regime which may be avoided if Dasatinib is used for senescence elimination clinically.

## 8 Senolytics and minority MOMP

Mitochondria are essential for cellular metabolism and respiration, but also apoptosis. The BCL-2 family are regulators of apoptosis and protect mitochondrial outer membrane integrity by binding to the pro‐apoptotic Bax and Bak, preventing pore formation and mitochondrial outer membrane permeabilization (MOMP) which allows activation of caspases which drives cells to apoptosis (Tait and Green, 2010). Recently, it was described that BH3 mimetics, including those related to navitoclax such as ABT‐737, can lead to incomplete MOMP in non-senescent cells, designated “minority MOMP” (Ichim et al., 2015). Limited caspase release as a result of minority MOMP is insufficient to trigger apoptosis but causes caspase‐dependent DNA damage and genomic instability (Ichim et al., 2015). Furthermore, minority MOMP is associated with a proinflammatory phenotype demonstrated by NF-κB activation, because of mitochondrial DNA release into the cytosol and activation of C-GAS-STING signalling (Riley et al., 2018). Therefore, while senolytics eliminate senescent cells, the long‐term effects on non-senescent tissues are unclear. In cancer cells, incomplete MOMP can result in genomic instability and resistance to anticancer therapies (Ichim et al., 2015; Liu et al., 2015). Additionally, it has been suggested that genome instability and inflammation associated with minority MOMP may drive premature senescence in the cell directly or in the surrounding tissues (Birch and Passos, 2017). Again, longer-term experiments are required, with the appropriate healthy controls to determine the systemic effects of senolytics augmenting apoptotic pathways.

## 9 Conclusions

**Fig. 6:**
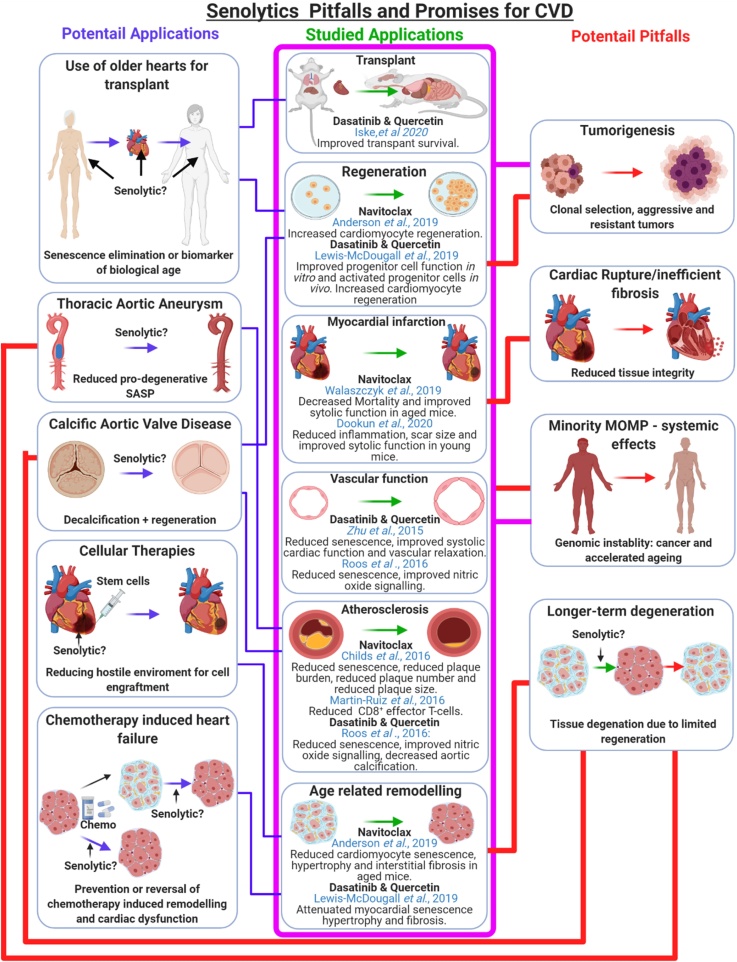
Summary of the promises and potential pitfalls for the clinical use of senotherapy for cardiovascular disease. Studies have demonstrated the potential for senolytics to attenuate the development or progression of CVD in multiple animal models (pink box). Based on these we hypothesise that senolytics may have further applications for CVD. These include improving heart transplantation outcome, preventing, or slowing the progression of TAA or CAVD, improving the survival and retention of cellular therapies via the elimination of the hostile environment created by senescence and the SASP in aged or infarcted hearts, and for the prevention or treatment of chemotherapy-induced cardiac dysfunction (blue connections). However, data from these previous studies also raised potential challenges that need to be addressed before senotherapy can be translated to the clinic. These include the potential promotion of TAA, valvular heart disease, cardiac rupture, or cardiomyopathy (red connections). All of these are associated with cell loss and may therefore arise as a result of the elimination of senescent cells, in the absence of a meaningful regenerative response. Furthermore, senolytic compounds may have detrimental systemic effects including tumorigenesis and minority MOMP-induced genomic instability increasing senescence and accelerated ageing (pink connections). Ultimately, this may further promote age-related CVD.

While obstacles and questions regarding possible detrimental influences and the longer-term effects of senolytics remain to be answered, the association between senescence and CVD, together with the enticing data from preclinical studies suggest that senotherapy has potential applications to prevent or reverse a wide range of CVDs and age-associated cardiovascular pathologies (Fig. 6). Animal studies are needed to specifically focus on the optimal timing of senolytic therapy, the potential adverse effects and whether the immediate benefits observed thus far are translated into a longer-term functional and/ or survival benefit. Interestingly, it has been suggested that while senescent cells can take months to develop, only 30 % of senescent cells need to be eliminated to see benefits in preclinical studies, as such a short-term hit and run approach to senolytic therapy may have the potential to improve tissue function in the long-term, while reducing detrimental side effects clinically (Hickson et al., 2019). Furthermore, targeted senolytics with fewer systemic effects are under development and will need to have their use in CVD explored, ideally rapidly moving on to clinical trials in this growing population.
